# Aqua­bis­(4-fluoro­benzoato-κ*O*)bis­(nicotinamide-κ*N* ^1^)copper(II) nicotinamide hemisolvate trihydrate

**DOI:** 10.1107/S1600536811053116

**Published:** 2011-12-17

**Authors:** Hacali Necefoğlu, Füreya Elif Özbek, Vijdan Öztürk, Vedat Adıgüzel, Tuncer Hökelek

**Affiliations:** a Kafkas University, Department of Chemistry, 36100 Kars, Turkey; b Hacettepe University, Department of Physics, 06800 Beytepe, Ankara, Turkey

## Abstract

The asymmetric unit of the title compound, [Cu(C_7_H_4_FO_2_)_2_(C_6_H_6_N_2_O)_2_(H_2_O)]·0.5C_6_H_6_N_2_O·3H_2_O, contains two aqua­bis­(4-fluoro­benzoato)bis­(nicotinamide)­copper(II) mol­ecules, one nicotinamide solvent mol­ecule and six water mol­ecules. The Cu^II^ ion is coordinated by two O atoms from two 4-fluoro­benzoate ligands, two N atoms from two nicotinamide ligands and one water O atom in a distorted square-pyramidal geometry. In the crystal, O—H⋯O, O—H⋯N and N—H⋯O hydrogen bonds consolidate the crystal packing, which also exhibits π–π inter­actions between the aromatic rings [centroid–centroid distances 3.692 (2)–3.794 (2) Å].

## Related literature

For general background to niacin, see: Krishnamachari (1974 ▶). For general background to the nicotinic acid derivative *N*,*N*-diethyl­nicotinamide, see: Bigoli *et al.* (1972 ▶). For related structures, see: Hökelek *et al.* (1996 ▶, 2009*a* ▶,*b* ▶); Hökelek & Necefoğlu (1998 ▶, 2007 ▶); Necefoğlu *et al.* (2011 ▶). For bond-length data, see: Allen *et al.* (1987 ▶).
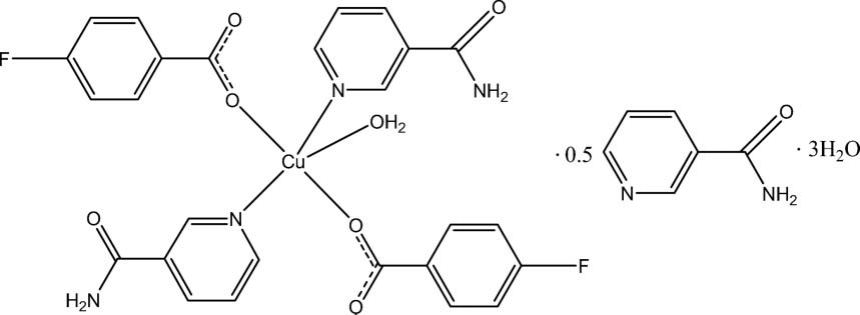


## Experimental

### 

#### Crystal data

[Cu(C_7_H_4_FO_2_)_2_(C_6_H_6_N_2_O)_2_(H_2_O)]·0.5C_6_H_6_N_2_O·3H_2_O
*M* *_r_* = 719.13
Monoclinic, 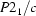

*a* = 18.4108 (4) Å
*b* = 14.8908 (3) Å
*c* = 22.8569 (5) Å
β = 105.247 (3)°
*V* = 6045.7 (2) Å^3^
*Z* = 8
Mo *K*α radiation
μ = 0.80 mm^−1^
*T* = 100 K
0.24 × 0.20 × 0.19 mm

#### Data collection

Bruker Kappa APEXII CCD area-detector diffractometer
Absorption correction: multi-scan (*SADABS*; Bruker, 2005 ▶) *T*_min_ = 0.825, *T*_max_ = 0.858
103729 measured reflections
15210 independent reflections
11162 reflections with *I* > 2σ(*I*)
*R*_int_ = 0.060

#### Refinement

*R*[*F* ^2^ > 2σ(*F* ^2^)] = 0.063
*wR*(*F* ^2^) = 0.160
*S* = 1.12
15210 reflections
900 parameters
34 restraints
H atoms treated by a mixture of independent and constrained refinement
Δρ_max_ = 1.27 e Å^−3^
Δρ_min_ = −1.16 e Å^−3^

### 

Data collection: *APEX2* (Bruker, 2007 ▶); cell refinement: *SAINT* (Bruker, 2007 ▶); data reduction: *SAINT*; program(s) used to solve structure: *SHELXS97* (Sheldrick, 2008 ▶); program(s) used to refine structure: *SHELXL97* (Sheldrick, 2008 ▶); molecular graphics: *ORTEP-3 for Windows* (Farrugia, 1997 ▶); software used to prepare material for publication: *WinGX* (Farrugia, 1999 ▶) and *PLATON* (Spek, 2009 ▶).

## Supplementary Material

Crystal structure: contains datablock(s) I, global. DOI: 10.1107/S1600536811053116/cv5200sup1.cif

Structure factors: contains datablock(s) I. DOI: 10.1107/S1600536811053116/cv5200Isup2.hkl

Additional supplementary materials:  crystallographic information; 3D view; checkCIF report

## Figures and Tables

**Table 1:** Hydrogen-bond geometry (Å, °)

*D*—H⋯*A*	*D*—H	H⋯*A*	*D*⋯*A*	*D*—H⋯*A*
N2—H2*A*⋯O18	0.86	2.10	2.929 (6)	163
N2—H2*B*⋯O4^i^	0.86	2.13	2.914 (5)	150
N4—H4*A*⋯O18^ii^	0.86	2.27	3.090 (6)	160
N4—H4*B*⋯O4^iii^	0.86	2.12	2.909 (5)	152
N6—H6*A*⋯O21^iv^	0.86	2.00	2.849 (5)	169
N6—H6*B*⋯O9^v^	0.86	2.18	2.925 (4)	145
N8—H8*A*⋯O16	0.86	2.44	3.285 (5)	167
N8—H8*B*⋯O9^vi^	0.86	2.07	2.890 (4)	160
N10—H10*A*⋯O7	0.86	2.20	3.031 (5)	163
N10—H10*B*⋯O12^vii^	0.86	2.10	2.897 (5)	155
O7—H71⋯O13^vii^	0.92 (3)	1.85 (3)	2.762 (4)	174 (3)
O7—H72⋯O14^vii^	0.81 (5)	2.02 (5)	2.824 (4)	174 (3)
O14—H141⋯N9	0.93 (4)	1.93 (4)	2.812 (4)	158 (4)
O14—H142⋯O5^viii^	0.93 (3)	1.85 (3)	2.782 (4)	177 (5)
O16—H161⋯O19	0.60 (4)	2.26 (3)	2.845 (6)	164 (8)
O17—H172⋯O6^ix^	0.73 (5)	2.21 (5)	2.887 (5)	156 (5)
O18—H182⋯O17^x^	0.63 (6)	2.30 (6)	2.839 (6)	145 (7)
O19—H191⋯O13	0.74 (5)	2.06 (5)	2.800 (6)	172 (5)
O20—H201⋯O18^viii^	0.77	2.07	2.611 (6)	128
O20—H202⋯O15	0.64	2.14	2.710 (5)	149
O21—H211⋯O2	0.91 (3)	1.91 (3)	2.807 (4)	169 (4)
O21—H212⋯O16^xi^	0.89 (4)	1.88 (5)	2.759 (5)	168 (5)

Symmetry codes: (i) 
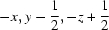
; (ii) 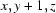; (iii) 
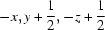
; (iv) 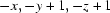; (v) 
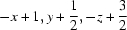
; (vi) 
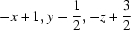
; (vii) 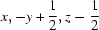; (viii) 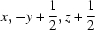; (ix) 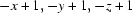; (x) 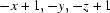; (xi) 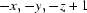.

**Fig. 1.**
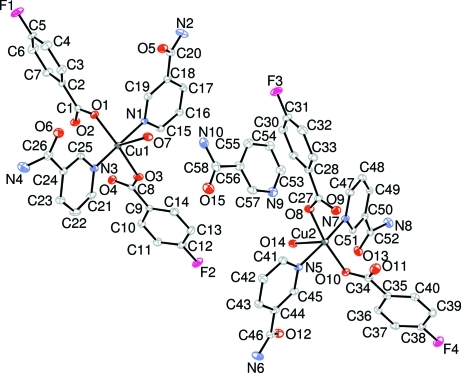
The content of asymmetric unit of the title compound showing the atomic numbering scheme and 50% probability displacement ellipsoids. Crystalline water molecules and hydrogen atoms have been omitted for clarity.

## supplementary crystallographic information

### Comment

As a part of our ongoing investigations of transition metal complexes of nicotinamide (NA), one form of niacin (Krishnamachari, 1974), and/or the nicotinic acid derivative *N*,*N*-diethylnicotinamide (DENA), an important respiratory stimulant (Bigoli *et al.*, 1972), the title compound was synthesized and its crystal structure is reported herein.

The asymmetric unit of the title mononuclear Cu^II^ complex (Fig. 1) contains two [Cu(*PFB*)_2_(*NA*)_2_(H_2_O)] molecules (*PFB* = 4-fluorobenzoato), one *NA* solvent molecule and six crystalline water molecules, all ligands coordinating in a monodentate manner. The crystal structures of similar omplexes of Cu^II^, Co^II^, Ni^II^, Mn^II^ and Zn^II^ ions, [Cu(C_7_H_5_O_2_)_2_(C_10_H_14_N_2_O)_2_] (Hökelek *et al.*, 1996), [Co(C_6_H_6_N_2_O)_2_(C_7_H_4_NO_4_)_2_(H_2_O)_2_] (Hökelek & Necefoğlu, 1998), [Co(C_9_H_9_O_2_)_2_(C_10_H_14_N_2_O)_2_(H_2_O)_2_] (Necefoğlu *et al.*, 2011), [Ni(C_7_H_4_ClO_2_)_2_(C_6_H_6_N_2_O)_2_(H_2_O)_2_] (Hökelek *et al.*, 2009*a*), [Mn(C_9_H_10_NO_2_)_2_(H_2_O)_4_].2H_2_O (Hökelek & Necefoğlu, 2007) and [Zn(C_7_H_4_BrO_2_)_2_(C_6_H_6_N_2_O)_2_(H_2_O)_2_] (Hökelek *et al.*, 2009*b*) have also been reported. In the copper(II) complex mentioned above the two benzoate ions coordinate to the Cu^II^ atom as bidentate ligands, while in the other structures all the ligands coordinate in a monodentate manner.

In the title compound, each Cu^II^ ion is coordinated by two O atoms from two *PFB* ligands, two N atoms from two *NA* ligands and one O_water_ atom in a distorted square-pyramidal geometry. The near equalities of the C1—O1 [1.281 (4) Å], C1—O2 [1.240 (4) Å], C8—O3 [1.282 (5) Å], C8—O4 [1.249 (5) Å] and C27—O8 [1.278 (4) Å], C27—O9 [1.249 (4) Å], C34—O10 [1.285 (5) Å], C34—O11 [1.245 (5) Å], bonds in the carboxylate groups indicate delocalized bonding arrangements, rather than localized single and double bonds. The Cu—O bond lengths are between 1.933 (3)-1.945 (3) Å (for benzoate oxygens) and 2.445 (3) Å and 2.479 (3) Å (for water oxygens), and the Cu—N bond lengths are between 2.021 (3)-2.044 (3) Å, close to standard values (Allen *et al.*, 1987). The intramolecular N—H···O, O—H···N and O—H···O hydrogen bonds (Table 1) link the water molecules to the nicotinamide ligands, carboxylate groups and to the uncoordianated water molecules. The Cu1 and Cu2 atoms are displaced out of the mean-planes of the adjacent carboxylate groups (O1/C1/O2), (O3/C8/O4) and (O8/C27/O9), (O10/C34/O11) by 0.1551 (4), -0.1910 (4) and -0.2732 (4), 0.4498 (4) Å, respectively. The dihedral angles between the planar carboxylate groups (O1/C1/O2), (O3/C8/O4) and (O8/C27/O9), (O10/C34/O11) and the adjacent benzene rings A (C2—C7), B (C9—C14) and E (C28—C33), F (C35—C40) are 11.34 (18), 14.87 (24) and 11.72 (18), 17.02 (21) °. The benzene and pyridine C (N1/C15—C19), D (N3/C21—C25) and G (N5/C41—C45), H(N7/C47—C51) rings are oriented at dihedral angles of A/B = 33.20 (13), A/C = 83.21 (13), A/D = 67.15 (13), B/C = 64.04 (13), B/D = 79.75 (13), C/D = 16.65 (11) and E/F = 45.16 (13), E/G = 76.73 (12), E/H = 58.51 (12), F/G = 58.75 (12), F/H = 76.90 (13), G/H = 18.23 (12) °.

In the crystal, intermolecular O—H···O and N—H···O hydrogen bonds (Table 1) link the molecules into a three-dimensional network. There also exist two weak C—H···π interactions (Table 1) and  the π–π contacts between the benzene and benzene rings and between the pyridine and pyridine rings Cg1—Cg1^i^, Cg1—Cg2^ii^, Cg2—Cg2^iii^, Cg5—Cg5^iv^, Cg5—Cg6^ii^, Cg6—Cg6^v^ and Cg3—Cg4^vi^, Cg7—Cg8^vii^, may further stabilize the structure [centroid-centroid distances = 3.851 (3), 3.846 (3), 3.869 (3), 3.888 (3), 3.756 (3), 3.990 (3) and 3.794 (2), 3.692 (2) Å; symmetry codes: (i)  -x, 1 - y, -z, (ii) x, 1/2 - y, -1/2 + z, (iii) -x, 1 - y, 1 - z, (iv) 1 - x, 1 - y, -z, (v) 1 - x, 1 - y, 1 - z, (vi) -x, -1/2 + y, 1/2 - z, (vii) 1 - x, -1/2 + y, 1/2 - z; Cg1, Cg2, Cg3, Cg4, Cg5, Cg6, Cg7 and Cg8 are the centroids of the rings A (C2—C7), B (C9—C14), C (N1/C15—C19), D (N3/C21—C25), E (C28—C33), F (C35—C40), G (N5/C41—C45) and H (N7/C47—C51), respectively.

### Experimental

The title compound was prepared by the reaction of CuSO_4_.5H_2_O (1.23 g, 5 mmol) in H_2_O (20 ml) and NA (1.22 g, 10 mmol) in H_2_O (20 ml) with sodium 4-fluorobenzoate (1.62 g, 10 mmol) in H_2_O (50 ml) at room temperature. The mixture was filtered and set aside to crystallize at ambient temperature for two weeks, giving blue single crystals.

### Refinement

Atoms H71, H72, H141, H142, H161, H162, H171, H172, H181, H182, H191, H192, H201, H202, H211 and H212 (for water molecules) were located in a difference Fourier map and were refined by applying restraints. The N-bound and C-bound H-atoms were positioned geometrically with N—H = 0.86 Å, for NH_2_ H-atoms, and C—H = 0.93 Å, for aromatic H-atoms, and constrained to ride on their parent atoms, with *U*_iso_(H) = 1.2 × *U*_eq_(C,N).

### Crystal data

**Table:**

[Cu(C_7_H_4_FO_2_)_2_(C_6_H_6_N_2_O)_2_(H_2_O)]·0.5C_6_H_6_N_2_O·3H_2_O	*F*(000) = 2968
*M**_r_* = 719.13	*D*_x_ = 1.580 Mg m^−^^3^
Monoclinic, *P*2_1_/*c*	Mo *K*α radiation, λ = 0.71073 Å
Hall symbol: -P 2ybc	Cell parameters from 9877 reflections
*a* = 18.4108 (4) Å	θ = 2.3–28.4°
*b* = 14.8908 (3) Å	µ = 0.80 mm^−^^1^
*c* = 22.8569 (5) Å	*T* = 100 K
β = 105.247 (3)°	Block, blue
*V* = 6045.7 (2) Å^3^	0.24 × 0.20 × 0.19 mm
*Z* = 8	

### Data collection

**Table:**

Bruker Kappa APEXII CCD area-detector diffractometer	15210 independent reflections
Radiation source: fine-focus sealed tube	11162 reflections with *I* > 2σ(*I*)
graphite	*R*_int_ = 0.060
φ and ω scans	θ_max_ = 28.5°, θ_min_ = 1.2°
Absorption correction: multi-scan (*SADABS*; Bruker, 2005)	*h* = −24→24
*T*_min_ = 0.825, *T*_max_ = 0.858	*k* = −17→19
103729 measured reflections	*l* = −30→29

### Refinement

**Table:**

Refinement on *F*^2^	Primary atom site location: structure-invariant direct methods
Least-squares matrix: full	Secondary atom site location: difference Fourier map
*R*[*F*^2^ > 2σ(*F*^2^)] = 0.063	Hydrogen site location: inferred from neighbouring sites
*wR*(*F*^2^) = 0.160	H atoms treated by a mixture of independent and constrained refinement
*S* = 1.12	*w* = 1/[σ^2^(*F*_o_^2^) + (0.0469*P*)^2^ + 20.8651*P*] where *P* = (*F*_o_^2^ + 2*F*_c_^2^)/3
15210 reflections	(Δ/σ)_max_ = 0.001
900 parameters	Δρ_max_ = 1.27 e Å^−^^3^
34 restraints	Δρ_min_ = −1.16 e Å^−^^3^

### Special details

**Table:**

Geometry. All esds (except the esd in the dihedral angle between two l.s. planes) are estimated using the full covariance matrix. The cell esds are taken into account individually in the estimation of esds in distances, angles and torsion angles; correlations between esds in cell parameters are only used when they are defined by crystal symmetry. An approximate (isotropic) treatment of cell esds is used for estimating esds involving l.s. planes.
Refinement. Refinement of F^2^ against ALL reflections. The weighted R-factor wR and goodness of fit S are based on F^2^, conventional R-factors R are based on F, with F set to zero for negative F^2^. The threshold expression of F^2^ > 2sigma(F^2^) is used only for calculating R-factors(gt) etc. and is not relevant to the choice of reflections for refinement. R-factors based on F^2^ are statistically about twice as large as those based on F, and R- factors based on ALL data will be even larger.

### Fractional atomic coordinates and isotropic or equivalent isotropic displacement parameters (Å^2^)

**Table:**

	*x*	*y*	*z*	*U*_iso_*/*U*_eq_	
Cu1	0.08147 (2)	0.39867 (3)	0.26867 (2)	0.01246 (11)	
Cu2	0.42670 (2)	0.18332 (3)	0.73175 (2)	0.01296 (11)	
O1	0.06780 (14)	0.39884 (18)	0.18189 (11)	0.0160 (5)	
O2	−0.05644 (15)	0.3901 (2)	0.16749 (12)	0.0201 (6)	
O3	0.08077 (16)	0.39915 (19)	0.35352 (12)	0.0198 (6)	
O4	−0.04449 (16)	0.40360 (19)	0.32462 (12)	0.0210 (6)	
O5	0.22275 (15)	0.1525 (2)	0.16971 (12)	0.0199 (6)	
O6	0.21882 (15)	0.6496 (2)	0.15711 (12)	0.0200 (6)	
O7	0.21840 (15)	0.3835 (2)	0.29314 (13)	0.0176 (6)	
H71	0.242 (2)	0.436 (2)	0.308 (2)	0.028 (13)*	
H72	0.236 (3)	0.362 (3)	0.2673 (18)	0.036 (15)*	
O8	0.43509 (14)	0.17369 (18)	0.64890 (12)	0.0163 (5)	
O9	0.55938 (14)	0.19444 (19)	0.67500 (12)	0.0181 (6)	
O10	0.43810 (15)	0.18487 (18)	0.81861 (12)	0.0170 (6)	
O11	0.56355 (16)	0.1867 (2)	0.83741 (13)	0.0245 (7)	
O12	0.28210 (15)	0.4238 (2)	0.82874 (13)	0.0211 (6)	
O13	0.28108 (15)	−0.0442 (2)	0.84207 (13)	0.0218 (6)	
O14	0.28719 (15)	0.18029 (19)	0.70437 (12)	0.0174 (6)	
H141	0.272 (3)	0.145 (3)	0.6696 (16)	0.044*	
H142	0.266 (3)	0.2367 (18)	0.694 (2)	0.044*	
O15	0.25627 (17)	0.3240 (2)	0.47017 (14)	0.0277 (7)	
O16	0.2515 (2)	−0.2231 (3)	0.9564 (2)	0.0412 (9)	
H161	0.239 (4)	−0.1857 (17)	0.956 (3)	0.044*	
H162	0.230 (3)	−0.214 (4)	0.972 (3)	0.044*	
O17	0.6950 (2)	0.2562 (3)	0.9124 (2)	0.0394 (9)	
H171	0.713 (4)	0.285 (3)	0.9312 (18)	0.044*	
H172	0.711 (3)	0.292 (3)	0.898 (2)	0.044*	
O18	0.2330 (2)	−0.0874 (3)	0.0866 (2)	0.0545 (12)	
H181	0.270 (3)	−0.104 (4)	0.113 (2)	0.044*	
H182	0.260 (3)	−0.112 (4)	0.084 (2)	0.044*	
O19	0.2134 (3)	−0.0382 (3)	0.9384 (2)	0.0525 (11)	
H191	0.230 (3)	−0.035 (4)	0.9120 (16)	0.044*	
H192	0.234 (3)	−0.001 (3)	0.958 (2)	0.044*	
O20	0.2792 (3)	0.4252 (3)	0.5720 (2)	0.0743 (15)	
H201	0.2682	0.4695	0.5547	0.044*	
H202	0.2624	0.4125	0.5450	0.044*	
O21	−0.21237 (17)	0.3687 (2)	0.12010 (14)	0.0279 (7)	
H211	−0.1621 (12)	0.380 (3)	0.131 (2)	0.044*	
H212	−0.218 (3)	0.322 (3)	0.095 (2)	0.044*	
N1	0.08483 (16)	0.2615 (2)	0.26844 (13)	0.0122 (6)	
N2	0.17473 (19)	0.0141 (2)	0.17423 (17)	0.0253 (8)	
H2A	0.1997	−0.0082	0.1508	0.030*	
H2B	0.1454	−0.0196	0.1883	0.030*	
N3	0.09809 (17)	0.5334 (2)	0.26912 (14)	0.0149 (6)	
N4	0.1643 (2)	0.7834 (2)	0.16339 (17)	0.0259 (8)	
H4A	0.1834	0.8072	0.1366	0.031*	
H4B	0.1359	0.8148	0.1800	0.031*	
N5	0.41525 (17)	0.3183 (2)	0.72846 (14)	0.0139 (6)	
N6	0.31899 (19)	0.5658 (2)	0.81972 (16)	0.0228 (8)	
H6A	0.2919	0.5863	0.8422	0.027*	
H6B	0.3457	0.6019	0.8047	0.027*	
N7	0.41236 (17)	0.0480 (2)	0.73400 (14)	0.0152 (6)	
N8	0.32489 (18)	−0.1853 (2)	0.84234 (16)	0.0214 (7)	
H8A	0.3018	−0.2027	0.8685	0.026*	
H8B	0.3518	−0.2227	0.8284	0.026*	
N9	0.25532 (19)	0.1124 (2)	0.58553 (16)	0.0221 (7)	
N10	0.24886 (19)	0.2368 (2)	0.38840 (16)	0.0239 (8)	
H10A	0.2493	0.2826	0.3656	0.029*	
H10B	0.2461	0.1835	0.3735	0.029*	
F1	−0.02454 (14)	0.3629 (2)	−0.10047 (10)	0.0292 (6)	
F2	0.01950 (14)	0.34751 (18)	0.60768 (10)	0.0266 (6)	
F3	0.49145 (14)	0.08908 (18)	0.39708 (10)	0.0270 (6)	
F4	0.52020 (13)	0.07895 (17)	1.09345 (10)	0.0240 (5)	
C1	0.0005 (2)	0.3913 (3)	0.14816 (17)	0.0147 (7)	
C2	−0.0058 (2)	0.3831 (3)	0.08151 (16)	0.0140 (7)	
C3	0.0562 (2)	0.4003 (3)	0.05915 (18)	0.0189 (8)	
H3	0.1019	0.4165	0.0856	0.023*	
C4	0.0500 (2)	0.3933 (3)	−0.00216 (18)	0.0234 (9)	
H4	0.0913	0.4043	−0.0174	0.028*	
C5	−0.0181 (2)	0.3697 (3)	−0.04024 (17)	0.0191 (8)	
C6	−0.0804 (2)	0.3515 (3)	−0.01972 (19)	0.0238 (9)	
H6	−0.1257	0.3343	−0.0464	0.029*	
C7	−0.0738 (2)	0.3594 (3)	0.04143 (18)	0.0208 (8)	
H7	−0.1155	0.3487	0.0561	0.025*	
C8	0.0162 (2)	0.3976 (3)	0.36483 (17)	0.0165 (8)	
C9	0.0169 (2)	0.3862 (3)	0.42988 (17)	0.0154 (7)	
C10	−0.0474 (2)	0.4021 (3)	0.44950 (18)	0.0185 (8)	
H10	−0.0912	0.4214	0.4218	0.022*	
C11	−0.0472 (2)	0.3895 (3)	0.50937 (18)	0.0188 (8)	
H11	−0.0901	0.4002	0.5225	0.023*	
C12	0.0184 (2)	0.3608 (3)	0.54878 (17)	0.0191 (8)	
C13	0.0833 (2)	0.3444 (3)	0.53159 (18)	0.0215 (9)	
H13	0.1267	0.3247	0.5595	0.026*	
C14	0.0822 (2)	0.3582 (3)	0.47177 (18)	0.0195 (8)	
H14	0.1257	0.3486	0.4593	0.023*	
C15	0.0631 (2)	0.2073 (3)	0.30758 (17)	0.0152 (7)	
H15	0.0392	0.2324	0.3348	0.018*	
C16	0.0748 (2)	0.1154 (3)	0.30898 (17)	0.0167 (8)	
H16	0.0576	0.0795	0.3359	0.020*	
C17	0.1122 (2)	0.0774 (3)	0.27013 (18)	0.0165 (8)	
H17	0.1207	0.0158	0.2704	0.020*	
C18	0.1370 (2)	0.1331 (3)	0.23057 (17)	0.0153 (7)	
C19	0.1215 (2)	0.2243 (3)	0.23078 (16)	0.0144 (7)	
H19	0.1372	0.2613	0.2037	0.017*	
C20	0.1814 (2)	0.1009 (3)	0.18868 (17)	0.0168 (8)	
C21	0.0810 (2)	0.5883 (3)	0.31046 (17)	0.0187 (8)	
H21	0.0599	0.5632	0.3394	0.022*	
C22	0.0930 (2)	0.6793 (3)	0.31204 (18)	0.0213 (8)	
H22	0.0806	0.7144	0.3416	0.026*	
C23	0.1238 (2)	0.7181 (3)	0.26920 (17)	0.0177 (8)	
H23	0.1317	0.7797	0.2689	0.021*	
C24	0.1426 (2)	0.6625 (3)	0.22630 (16)	0.0138 (7)	
C25	0.1293 (2)	0.5711 (3)	0.22823 (16)	0.0132 (7)	
H25	0.1426	0.5341	0.1999	0.016*	
C26	0.1785 (2)	0.6982 (3)	0.17921 (17)	0.0162 (8)	
C27	0.4991 (2)	0.1754 (3)	0.63700 (17)	0.0152 (7)	
C28	0.4987 (2)	0.1499 (3)	0.57344 (17)	0.0147 (7)	
C29	0.4341 (2)	0.1129 (3)	0.53510 (17)	0.0174 (8)	
H29	0.3922	0.1023	0.5496	0.021*	
C30	0.4315 (2)	0.0917 (3)	0.47550 (17)	0.0197 (8)	
H30	0.3886	0.0667	0.4498	0.024*	
C31	0.4944 (2)	0.1088 (3)	0.45565 (17)	0.0200 (8)	
C32	0.5601 (2)	0.1439 (3)	0.49182 (19)	0.0204 (8)	
H32	0.6019	0.1536	0.4770	0.024*	
C33	0.5614 (2)	0.1642 (3)	0.55158 (18)	0.0177 (8)	
H33	0.6050	0.1877	0.5773	0.021*	
C34	0.5048 (2)	0.1770 (3)	0.85397 (17)	0.0177 (8)	
C35	0.5083 (2)	0.1522 (3)	0.91824 (17)	0.0162 (8)	
C36	0.4457 (2)	0.1608 (3)	0.94108 (17)	0.0166 (8)	
H36	0.4007	0.1821	0.9161	0.020*	
C37	0.4497 (2)	0.1380 (3)	1.00013 (18)	0.0191 (8)	
H37	0.4084	0.1459	1.0159	0.023*	
C38	0.5160 (2)	0.1036 (3)	1.03528 (17)	0.0177 (8)	
C39	0.5794 (2)	0.0924 (3)	1.01446 (18)	0.0178 (8)	
H39	0.6236	0.0688	1.0393	0.021*	
C40	0.5748 (2)	0.1175 (3)	0.95534 (17)	0.0158 (7)	
H40	0.6167	0.1112	0.9402	0.019*	
C41	0.4338 (2)	0.3706 (3)	0.68716 (17)	0.0169 (8)	
H41	0.4563	0.3443	0.6594	0.020*	
C42	0.4208 (2)	0.4623 (3)	0.68388 (18)	0.0186 (8)	
H42	0.4356	0.4969	0.6552	0.022*	
C43	0.3853 (2)	0.5017 (3)	0.72407 (17)	0.0181 (8)	
H43	0.3760	0.5632	0.7228	0.022*	
C44	0.3640 (2)	0.4473 (3)	0.76622 (17)	0.0160 (8)	
C45	0.3807 (2)	0.3564 (3)	0.76707 (16)	0.0148 (7)	
H45	0.3672	0.3201	0.7957	0.018*	
C46	0.3194 (2)	0.4785 (3)	0.80826 (17)	0.0165 (8)	
C47	0.4298 (2)	−0.0115 (3)	0.69489 (17)	0.0177 (8)	
H47	0.4527	0.0099	0.6659	0.021*	
C48	0.4153 (2)	−0.1015 (3)	0.69599 (18)	0.0197 (8)	
H48	0.4291	−0.1401	0.6688	0.024*	
C49	0.3800 (2)	−0.1346 (3)	0.73790 (17)	0.0158 (7)	
H49	0.3695	−0.1955	0.7393	0.019*	
C50	0.3606 (2)	−0.0748 (3)	0.77775 (17)	0.0153 (7)	
C51	0.37847 (19)	0.0150 (3)	0.77445 (16)	0.0141 (7)	
H51	0.3662	0.0546	0.8019	0.017*	
C52	0.3191 (2)	−0.1009 (3)	0.82351 (18)	0.0170 (8)	
C53	0.2484 (2)	0.0285 (3)	0.56354 (19)	0.0226 (9)	
H53	0.2476	−0.0189	0.5899	0.027*	
C54	0.2425 (2)	0.0093 (3)	0.50311 (18)	0.0210 (8)	
H54	0.2387	−0.0498	0.4896	0.025*	
C55	0.2424 (2)	0.0792 (3)	0.46307 (19)	0.0217 (9)	
H55	0.2374	0.0676	0.4222	0.026*	
C56	0.2497 (2)	0.1665 (3)	0.48470 (19)	0.0205 (8)	
C57	0.2559 (2)	0.1792 (3)	0.54637 (19)	0.0210 (8)	
H57	0.2608	0.2377	0.5612	0.025*	
C58	0.2523 (2)	0.2486 (3)	0.4470 (2)	0.0225 (9)	

### Atomic displacement parameters (Å^2^)

**Table:**

	*U*^11^	*U*^22^	*U*^33^	*U*^12^	*U*^13^	*U*^23^
Cu1	0.0153 (2)	0.0130 (2)	0.0107 (2)	0.00078 (17)	0.00629 (16)	0.00059 (18)
Cu2	0.0144 (2)	0.0151 (2)	0.0107 (2)	−0.00007 (17)	0.00571 (16)	−0.00086 (18)
O1	0.0191 (13)	0.0169 (14)	0.0115 (12)	−0.0002 (11)	0.0030 (10)	−0.0009 (11)
O2	0.0217 (14)	0.0263 (16)	0.0149 (13)	0.0016 (12)	0.0094 (11)	−0.0002 (12)
O3	0.0274 (15)	0.0200 (15)	0.0165 (13)	0.0022 (12)	0.0134 (11)	−0.0005 (11)
O4	0.0280 (15)	0.0206 (15)	0.0145 (13)	0.0017 (12)	0.0054 (11)	0.0019 (11)
O5	0.0171 (13)	0.0259 (16)	0.0180 (14)	−0.0019 (11)	0.0068 (11)	−0.0040 (12)
O6	0.0187 (13)	0.0249 (16)	0.0196 (14)	0.0030 (11)	0.0108 (11)	0.0013 (12)
O7	0.0162 (13)	0.0193 (15)	0.0195 (14)	−0.0013 (11)	0.0085 (11)	−0.0032 (12)
O8	0.0166 (13)	0.0186 (14)	0.0164 (13)	−0.0018 (11)	0.0089 (10)	−0.0028 (11)
O9	0.0157 (13)	0.0222 (15)	0.0170 (13)	−0.0010 (11)	0.0054 (10)	−0.0025 (11)
O10	0.0197 (13)	0.0184 (14)	0.0122 (12)	0.0014 (11)	0.0027 (10)	−0.0019 (11)
O11	0.0236 (15)	0.0340 (18)	0.0182 (14)	−0.0015 (13)	0.0098 (12)	0.0034 (13)
O12	0.0209 (14)	0.0231 (16)	0.0228 (15)	−0.0032 (12)	0.0119 (12)	−0.0042 (12)
O13	0.0214 (14)	0.0242 (16)	0.0239 (15)	0.0055 (12)	0.0131 (12)	0.0057 (12)
O14	0.0180 (13)	0.0182 (15)	0.0174 (13)	0.0007 (11)	0.0071 (11)	−0.0026 (11)
O15	0.0281 (16)	0.0247 (17)	0.0328 (17)	0.0000 (13)	0.0123 (13)	0.0001 (14)
O16	0.040 (2)	0.034 (2)	0.054 (3)	−0.0011 (18)	0.0187 (18)	−0.0092 (19)
O17	0.040 (2)	0.040 (2)	0.042 (2)	−0.0064 (17)	0.0167 (18)	0.0105 (18)
O18	0.043 (2)	0.059 (3)	0.069 (3)	0.007 (2)	0.027 (2)	−0.018 (2)
O19	0.072 (3)	0.051 (3)	0.050 (3)	0.010 (2)	0.044 (2)	0.009 (2)
O20	0.084 (4)	0.074 (4)	0.067 (3)	−0.018 (3)	0.025 (3)	−0.007 (3)
O21	0.0235 (15)	0.0342 (19)	0.0292 (17)	0.0078 (14)	0.0127 (13)	−0.0018 (14)
N1	0.0138 (14)	0.0136 (16)	0.0098 (14)	0.0004 (12)	0.0044 (11)	0.0000 (12)
N2	0.0215 (17)	0.0211 (19)	0.036 (2)	−0.0017 (14)	0.0130 (15)	−0.0129 (16)
N3	0.0147 (15)	0.0164 (17)	0.0146 (15)	0.0005 (12)	0.0057 (12)	−0.0011 (13)
N4	0.038 (2)	0.0177 (18)	0.031 (2)	−0.0012 (16)	0.0239 (17)	0.0020 (15)
N5	0.0141 (14)	0.0155 (16)	0.0132 (15)	−0.0022 (12)	0.0057 (12)	−0.0020 (13)
N6	0.0244 (18)	0.0207 (19)	0.0290 (19)	−0.0005 (14)	0.0173 (15)	−0.0058 (15)
N7	0.0133 (14)	0.0209 (17)	0.0125 (15)	0.0016 (13)	0.0053 (12)	−0.0017 (13)
N8	0.0205 (16)	0.0224 (19)	0.0272 (18)	0.0018 (14)	0.0165 (14)	0.0060 (15)
N9	0.0206 (17)	0.0243 (19)	0.0238 (18)	−0.0021 (14)	0.0099 (14)	−0.0004 (15)
N10	0.0273 (18)	0.0180 (18)	0.0258 (19)	0.0023 (14)	0.0063 (15)	0.0045 (15)
F1	0.0254 (13)	0.0538 (18)	0.0112 (11)	−0.0005 (12)	0.0097 (9)	−0.0023 (11)
F2	0.0291 (13)	0.0410 (16)	0.0115 (11)	−0.0004 (11)	0.0087 (10)	0.0009 (11)
F3	0.0334 (14)	0.0382 (16)	0.0124 (11)	0.0017 (11)	0.0109 (10)	−0.0032 (10)
F4	0.0273 (12)	0.0346 (15)	0.0124 (11)	−0.0016 (11)	0.0091 (9)	0.0040 (10)
C1	0.0189 (18)	0.0104 (18)	0.0155 (18)	0.0002 (14)	0.0057 (14)	0.0016 (14)
C2	0.0180 (17)	0.0128 (18)	0.0119 (17)	0.0001 (14)	0.0053 (14)	0.0021 (14)
C3	0.0154 (17)	0.024 (2)	0.0183 (19)	−0.0029 (16)	0.0059 (15)	0.0003 (16)
C4	0.0211 (19)	0.033 (2)	0.019 (2)	−0.0036 (18)	0.0116 (16)	−0.0003 (18)
C5	0.025 (2)	0.025 (2)	0.0087 (17)	0.0033 (17)	0.0077 (15)	0.0004 (15)
C6	0.0159 (18)	0.037 (3)	0.019 (2)	−0.0028 (17)	0.0046 (15)	−0.0012 (18)
C7	0.0185 (19)	0.030 (2)	0.0167 (19)	−0.0016 (17)	0.0090 (15)	−0.0013 (17)
C8	0.029 (2)	0.0083 (18)	0.0147 (18)	0.0021 (15)	0.0103 (15)	0.0004 (14)
C9	0.0215 (18)	0.0122 (18)	0.0137 (17)	0.0012 (14)	0.0068 (14)	−0.0011 (14)
C10	0.0178 (18)	0.020 (2)	0.0175 (18)	0.0034 (15)	0.0048 (15)	−0.0006 (16)
C11	0.0170 (18)	0.022 (2)	0.0196 (19)	−0.0010 (15)	0.0090 (15)	−0.0007 (16)
C12	0.026 (2)	0.021 (2)	0.0121 (18)	−0.0019 (16)	0.0085 (15)	−0.0007 (15)
C13	0.0208 (19)	0.028 (2)	0.0157 (19)	0.0051 (17)	0.0048 (15)	0.0025 (17)
C14	0.0210 (19)	0.022 (2)	0.0185 (19)	0.0007 (16)	0.0103 (15)	−0.0012 (16)
C15	0.0177 (17)	0.0150 (19)	0.0147 (18)	−0.0011 (14)	0.0075 (14)	−0.0016 (15)
C16	0.0221 (19)	0.0145 (19)	0.0155 (18)	−0.0012 (15)	0.0085 (15)	0.0014 (15)
C17	0.0166 (17)	0.0113 (18)	0.0216 (19)	0.0006 (14)	0.0052 (15)	−0.0005 (15)
C18	0.0122 (16)	0.019 (2)	0.0143 (17)	0.0000 (14)	0.0028 (14)	−0.0020 (15)
C19	0.0138 (17)	0.0172 (19)	0.0122 (17)	−0.0020 (14)	0.0037 (13)	−0.0004 (14)
C20	0.0102 (16)	0.022 (2)	0.0171 (18)	−0.0001 (15)	0.0026 (14)	−0.0041 (16)
C21	0.0227 (19)	0.023 (2)	0.0122 (17)	−0.0046 (16)	0.0088 (15)	−0.0015 (15)
C22	0.028 (2)	0.021 (2)	0.019 (2)	−0.0034 (17)	0.0139 (16)	−0.0053 (17)
C23	0.0198 (18)	0.016 (2)	0.0180 (19)	−0.0019 (15)	0.0054 (15)	−0.0041 (15)
C24	0.0136 (16)	0.0150 (19)	0.0131 (17)	−0.0008 (14)	0.0042 (13)	0.0005 (14)
C25	0.0136 (16)	0.0154 (19)	0.0119 (17)	0.0019 (14)	0.0057 (13)	−0.0001 (14)
C26	0.0128 (16)	0.020 (2)	0.0153 (18)	−0.0040 (15)	0.0025 (14)	−0.0004 (15)
C27	0.0191 (18)	0.0137 (19)	0.0142 (18)	0.0001 (14)	0.0069 (14)	−0.0002 (14)
C28	0.0201 (18)	0.0124 (18)	0.0134 (17)	0.0015 (14)	0.0072 (14)	−0.0008 (14)
C29	0.0188 (18)	0.017 (2)	0.0186 (19)	0.0007 (15)	0.0088 (15)	0.0000 (15)
C30	0.0232 (19)	0.022 (2)	0.0138 (18)	0.0005 (16)	0.0053 (15)	−0.0004 (16)
C31	0.028 (2)	0.023 (2)	0.0121 (18)	0.0041 (17)	0.0110 (15)	0.0018 (16)
C32	0.023 (2)	0.020 (2)	0.024 (2)	0.0022 (16)	0.0150 (16)	0.0030 (17)
C33	0.0196 (18)	0.016 (2)	0.0197 (19)	−0.0004 (15)	0.0090 (15)	−0.0006 (15)
C34	0.0236 (19)	0.0150 (19)	0.0157 (18)	0.0010 (15)	0.0076 (15)	−0.0017 (15)
C35	0.0182 (18)	0.0158 (19)	0.0151 (18)	−0.0008 (15)	0.0052 (14)	−0.0022 (15)
C36	0.0174 (18)	0.0140 (19)	0.0189 (19)	0.0021 (14)	0.0057 (15)	−0.0006 (15)
C37	0.0193 (19)	0.021 (2)	0.0197 (19)	−0.0003 (16)	0.0105 (15)	−0.0043 (16)
C38	0.0230 (19)	0.020 (2)	0.0104 (17)	−0.0021 (16)	0.0045 (14)	−0.0009 (15)
C39	0.0188 (18)	0.017 (2)	0.0176 (19)	−0.0002 (15)	0.0044 (15)	0.0014 (15)
C40	0.0159 (17)	0.0143 (19)	0.0190 (18)	−0.0020 (14)	0.0075 (14)	−0.0026 (15)
C41	0.0184 (18)	0.017 (2)	0.0178 (18)	0.0002 (15)	0.0085 (15)	−0.0001 (15)
C42	0.0209 (19)	0.017 (2)	0.0204 (19)	0.0002 (15)	0.0106 (15)	0.0024 (16)
C43	0.0179 (18)	0.018 (2)	0.0200 (19)	0.0025 (15)	0.0070 (15)	−0.0016 (16)
C44	0.0112 (16)	0.023 (2)	0.0148 (18)	−0.0029 (14)	0.0051 (14)	−0.0044 (15)
C45	0.0132 (17)	0.019 (2)	0.0131 (17)	−0.0025 (14)	0.0056 (13)	−0.0028 (15)
C46	0.0100 (16)	0.023 (2)	0.0161 (18)	−0.0015 (15)	0.0034 (14)	−0.0052 (16)
C47	0.0168 (18)	0.023 (2)	0.0156 (18)	0.0024 (15)	0.0080 (14)	−0.0021 (16)
C48	0.0231 (19)	0.022 (2)	0.0162 (18)	0.0056 (16)	0.0084 (15)	−0.0005 (16)
C49	0.0174 (18)	0.0138 (19)	0.0156 (18)	0.0033 (14)	0.0036 (14)	0.0017 (15)
C50	0.0124 (16)	0.019 (2)	0.0141 (17)	0.0000 (14)	0.0028 (14)	0.0023 (15)
C51	0.0131 (16)	0.019 (2)	0.0107 (16)	0.0025 (14)	0.0041 (13)	0.0009 (14)
C52	0.0120 (16)	0.020 (2)	0.0196 (19)	0.0014 (15)	0.0045 (14)	0.0027 (16)
C53	0.0204 (19)	0.024 (2)	0.024 (2)	−0.0021 (17)	0.0071 (16)	0.0005 (17)
C55	0.0173 (18)	0.029 (2)	0.021 (2)	−0.0020 (16)	0.0082 (16)	−0.0010 (17)
C54	0.0220 (19)	0.021 (2)	0.022 (2)	−0.0030 (16)	0.0081 (16)	0.0006 (17)
C56	0.0152 (18)	0.024 (2)	0.024 (2)	−0.0010 (16)	0.0092 (15)	−0.0016 (17)
C57	0.0175 (18)	0.024 (2)	0.024 (2)	0.0013 (16)	0.0107 (16)	−0.0026 (17)
C58	0.0130 (18)	0.027 (2)	0.028 (2)	0.0033 (16)	0.0069 (16)	0.0009 (18)

### Geometric parameters (Å, °)

**Table:**

Cu1—O1	1.933 (3)	C12—C11	1.371 (6)
Cu1—O3	1.943 (3)	C12—C13	1.375 (6)
Cu1—N1	2.044 (3)	C13—C14	1.377 (5)
Cu1—N3	2.030 (3)	C13—H13	0.9300
Cu2—O8	1.945 (3)	C14—H14	0.9300
Cu2—O10	1.941 (3)	C15—C16	1.384 (5)
Cu2—N5	2.021 (3)	C15—H15	0.9300
Cu2—N7	2.034 (3)	C16—H16	0.9300
O1—C1	1.281 (4)	C17—C16	1.380 (5)
O2—C1	1.240 (4)	C17—H17	0.9300
O3—C8	1.282 (5)	C18—C17	1.389 (5)
O4—C8	1.249 (5)	C18—C20	1.492 (5)
O5—C20	1.237 (5)	C19—C18	1.388 (5)
O6—C26	1.235 (5)	C19—H19	0.9300
O7—H71	0.917 (18)	C20—N2	1.332 (5)
O7—H72	0.81 (2)	C21—H21	0.9300
O8—C27	1.278 (4)	C22—C21	1.371 (6)
O9—C27	1.249 (4)	C22—H22	0.9300
O10—C34	1.285 (5)	C23—C22	1.381 (5)
O11—C34	1.245 (5)	C23—H23	0.9300
O12—C46	1.235 (5)	C24—C23	1.395 (5)
O13—C52	1.241 (5)	C24—C25	1.386 (5)
O14—H141	0.93 (2)	C25—H25	0.9300
O14—H142	0.930 (18)	C26—N4	1.326 (5)
O15—C58	1.237 (5)	C26—C24	1.501 (5)
O16—H161	0.601 (8)	C27—C28	1.499 (5)
O16—H162	0.618 (11)	C28—C33	1.389 (5)
O17—H171	0.633 (8)	C29—C30	1.387 (5)
O17—H172	0.73 (2)	C29—C28	1.393 (5)
O18—H181	0.812 (18)	C29—H29	0.9300
O18—H182	0.64 (2)	C30—H30	0.9300
O19—H191	0.745 (18)	C31—C30	1.374 (6)
O19—H192	0.742 (18)	C32—C31	1.376 (6)
O20—H201	0.770	C32—C33	1.393 (5)
O20—H202	0.640	C32—H32	0.9300
O21—H211	0.911 (18)	C33—H33	0.9300
O21—H212	0.90 (2)	C34—C35	1.499 (5)
N1—C15	1.342 (5)	C35—C40	1.391 (5)
N1—C19	1.345 (5)	C36—C37	1.375 (5)
N2—H2A	0.8600	C36—C35	1.391 (5)
N2—H2B	0.8600	C36—H36	0.9300
N3—C21	1.348 (5)	C37—H37	0.9300
N3—C25	1.342 (5)	C38—C37	1.371 (5)
N4—H4A	0.8600	C38—C39	1.382 (5)
N4—H4B	0.8600	C39—H39	0.9300
N5—C41	1.335 (5)	C40—C39	1.383 (5)
N5—C45	1.342 (5)	C40—H40	0.9300
N6—C46	1.326 (5)	C41—C42	1.385 (6)
N6—H6A	0.8600	C41—H41	0.9300
N6—H6B	0.8600	C42—H42	0.9300
N7—C51	1.337 (5)	C43—C42	1.390 (5)
N7—C47	1.356 (5)	C43—H43	0.9300
N8—C52	1.322 (5)	C44—C43	1.391 (5)
N8—H8A	0.8600	C44—C46	1.493 (5)
N8—H8B	0.8600	C45—C44	1.388 (6)
N10—H10A	0.8600	C45—H45	0.9300
N10—H10B	0.8600	C47—C48	1.368 (6)
F1—C5	1.354 (4)	C47—H47	0.9300
F2—C12	1.355 (4)	C48—C49	1.382 (5)
F3—C31	1.358 (4)	C48—H48	0.9300
F4—C38	1.361 (4)	C49—H49	0.9300
C1—C2	1.502 (5)	C50—C51	1.384 (5)
C2—C3	1.392 (5)	C50—C49	1.386 (5)
C2—C7	1.388 (5)	C50—C52	1.501 (5)
C3—C4	1.380 (5)	C51—H51	0.9300
C3—H3	0.9300	C53—N9	1.341 (6)
C4—C5	1.371 (6)	C53—C54	1.386 (6)
C4—H4	0.9300	C53—H53	0.9300
C6—C5	1.376 (6)	C54—H54	0.9300
C6—C7	1.376 (6)	C55—C54	1.385 (6)
C6—H6	0.9300	C55—C56	1.384 (6)
C7—H7	0.9300	C55—H55	0.9300
C8—C9	1.493 (5)	C57—N9	1.340 (5)
C9—C10	1.392 (5)	C57—C56	1.397 (6)
C9—C14	1.388 (5)	C57—H57	0.9300
C10—C11	1.380 (5)	C58—N10	1.334 (5)
C10—H10	0.9300	C58—C56	1.504 (6)
C11—H11	0.9300		
			
O1—Cu1—O3	172.41 (12)	N3—C21—H21	118.3
O1—Cu1—N1	89.69 (12)	C22—C21—H21	118.3
O1—Cu1—N3	89.01 (12)	C21—C22—C23	119.2 (4)
O3—Cu1—N1	90.82 (12)	C21—C22—H22	120.4
O3—Cu1—N3	91.82 (12)	C23—C22—H22	120.4
N3—Cu1—N1	169.64 (12)	C22—C23—C24	118.3 (4)
O8—Cu2—N5	94.10 (12)	C22—C23—H23	120.8
O8—Cu2—N7	89.75 (12)	C24—C23—H23	120.8
O10—Cu2—O8	169.03 (11)	C23—C24—C26	122.1 (3)
O10—Cu2—N5	90.48 (12)	C25—C24—C23	118.8 (3)
O10—Cu2—N7	88.04 (12)	C25—C24—C26	119.1 (3)
N5—Cu2—N7	166.71 (12)	N3—C25—C24	123.0 (3)
C1—O1—Cu1	117.5 (2)	N3—C25—H25	118.5
C8—O3—Cu1	116.8 (2)	C24—C25—H25	118.5
H72—O7—H71	110 (4)	O6—C26—N4	123.1 (4)
C27—O8—Cu2	121.3 (2)	O6—C26—C24	120.7 (4)
C34—O10—Cu2	118.1 (2)	N4—C26—C24	116.2 (3)
H142—O14—H141	106 (3)	O8—C27—C28	115.6 (3)
H161—O16—H162	60 (9)	O9—C27—O8	123.9 (3)
H171—O17—H172	67 (5)	O9—C27—C28	120.6 (3)
H181—O18—H182	51 (4)	C29—C28—C27	119.7 (3)
H191—O19—H192	100 (4)	C33—C28—C27	121.1 (3)
H201—O20—H202	76	C33—C28—C29	119.2 (3)
H212—O21—H211	106 (4)	C28—C29—H29	119.6
C15—N1—Cu1	125.8 (3)	C30—C29—C28	120.7 (4)
C15—N1—C19	117.9 (3)	C30—C29—H29	119.6
C19—N1—Cu1	115.7 (2)	C29—C30—H30	121.0
C20—N2—H2A	120.0	C31—C30—C29	117.9 (4)
C20—N2—H2B	120.0	C31—C30—H30	121.0
H2A—N2—H2B	120.0	F3—C31—C30	117.8 (4)
C21—N3—Cu1	122.7 (3)	F3—C31—C32	118.4 (3)
C25—N3—Cu1	120.1 (3)	C30—C31—C32	123.8 (4)
C25—N3—C21	117.2 (3)	C31—C32—C33	117.2 (4)
C26—N4—H4A	120.0	C31—C32—H32	121.4
C26—N4—H4B	120.0	C33—C32—H32	121.4
H4A—N4—H4B	120.0	C28—C33—C32	121.2 (4)
C41—N5—Cu2	124.2 (3)	C28—C33—H33	119.4
C41—N5—C45	118.2 (3)	C32—C33—H33	119.4
C45—N5—Cu2	117.3 (3)	O10—C34—C35	115.3 (3)
C46—N6—H6A	120.0	O11—C34—O10	124.1 (4)
C46—N6—H6B	120.0	O11—C34—C35	120.6 (3)
H6A—N6—H6B	120.0	C36—C35—C34	121.2 (3)
C47—N7—Cu2	125.1 (3)	C36—C35—C40	119.2 (4)
C51—N7—Cu2	117.9 (3)	C40—C35—C34	119.6 (3)
C51—N7—C47	116.8 (3)	C35—C36—H36	119.6
C52—N8—H8A	120.0	C37—C36—C35	120.7 (4)
C52—N8—H8B	120.0	C37—C36—H36	119.6
H8A—N8—H8B	120.0	C36—C37—H37	120.8
C57—N9—C53	117.3 (4)	C38—C37—C36	118.5 (4)
C58—N10—H10A	120.0	C38—C37—H37	120.8
C58—N10—H10B	120.0	F4—C38—C37	119.1 (3)
H10A—N10—H10B	120.0	F4—C38—C39	117.9 (3)
O1—C1—C2	115.0 (3)	C37—C38—C39	123.0 (4)
O2—C1—O1	124.2 (3)	C38—C39—C40	117.6 (4)
O2—C1—C2	120.8 (3)	C38—C39—H39	121.2
C3—C2—C1	120.4 (3)	C40—C39—H39	121.2
C7—C2—C1	120.4 (3)	C35—C40—H40	119.5
C7—C2—C3	119.2 (3)	C39—C40—C35	120.9 (4)
C2—C3—H3	119.9	C39—C40—H40	119.5
C4—C3—C2	120.2 (4)	N5—C41—C42	122.7 (4)
C4—C3—H3	119.9	N5—C41—H41	118.6
C3—C4—H4	120.6	C42—C41—H41	118.6
C5—C4—C3	118.7 (4)	C41—C42—C43	119.0 (4)
C5—C4—H4	120.6	C41—C42—H42	120.5
F1—C5—C4	119.1 (3)	C43—C42—H42	120.5
F1—C5—C6	118.3 (4)	C42—C43—C44	118.6 (4)
C4—C5—C6	122.7 (4)	C42—C43—H43	120.7
C5—C6—C7	118.1 (4)	C44—C43—H43	120.7
C5—C6—H6	121.0	C43—C44—C46	124.6 (4)
C7—C6—H6	121.0	C45—C44—C43	118.5 (3)
C2—C7—H7	119.5	C45—C44—C46	116.8 (3)
C6—C7—C2	121.1 (4)	N5—C45—C44	122.9 (4)
C6—C7—H7	119.5	N5—C45—H45	118.5
O3—C8—C9	115.9 (3)	C44—C45—H45	118.5
O4—C8—O3	123.2 (3)	O12—C46—N6	122.6 (4)
O4—C8—C9	120.9 (3)	O12—C46—C44	119.6 (4)
C10—C9—C8	121.0 (3)	N6—C46—C44	117.7 (4)
C14—C9—C8	120.1 (3)	N7—C47—C48	123.2 (4)
C14—C9—C10	118.9 (3)	N7—C47—H47	118.4
C9—C10—H10	119.5	C48—C47—H47	118.4
C11—C10—C9	121.0 (4)	C47—C48—C49	119.4 (4)
C11—C10—H10	119.5	C47—C48—H48	120.3
C10—C11—H11	121.1	C49—C48—H48	120.3
C12—C11—C10	117.8 (4)	C48—C49—C50	118.4 (4)
C12—C11—H11	121.1	C48—C49—H49	120.8
F2—C12—C11	118.6 (3)	C50—C49—H49	120.8
F2—C12—C13	118.1 (4)	C51—C50—C49	118.7 (3)
C11—C12—C13	123.3 (4)	C51—C50—C52	117.4 (3)
C12—C13—C14	118.0 (4)	C49—C50—C52	123.9 (4)
C12—C13—H13	121.0	N7—C51—C50	123.6 (4)
C14—C13—H13	121.0	N7—C51—H51	118.2
C9—C14—H14	119.5	C50—C51—H51	118.2
C13—C14—C9	120.9 (4)	O13—C52—N8	122.7 (4)
C13—C14—H14	119.5	O13—C52—C50	119.9 (4)
N1—C15—C16	122.5 (3)	N8—C52—C50	117.4 (3)
N1—C15—H15	118.7	N9—C53—C54	122.7 (4)
C16—C15—H15	118.7	N9—C53—H53	118.7
C15—C16—H16	120.3	C54—C53—H53	118.7
C17—C16—C15	119.4 (4)	C53—C54—H54	120.3
C17—C16—H16	120.3	C55—C54—C53	119.3 (4)
C16—C17—C18	118.6 (4)	C55—C54—H54	120.3
C16—C17—H17	120.7	C54—C55—H55	120.5
C18—C17—H17	120.7	C56—C55—C54	119.1 (4)
C17—C18—C20	123.7 (4)	C56—C55—H55	120.5
C19—C18—C17	118.6 (3)	C55—C56—C57	117.5 (4)
C19—C18—C20	117.7 (3)	C55—C56—C58	125.0 (4)
N1—C19—C18	122.9 (3)	C57—C56—C58	117.5 (4)
N1—C19—H19	118.5	N9—C57—C56	124.1 (4)
C18—C19—H19	118.5	N9—C57—H57	118.0
O5—C20—N2	122.8 (4)	C56—C57—H57	118.0
O5—C20—C18	121.1 (4)	O15—C58—N10	122.1 (4)
N2—C20—C18	116.1 (4)	O15—C58—C56	120.0 (4)
N3—C21—C22	123.5 (4)	N10—C58—C56	117.9 (4)
			
N1—Cu1—O1—C1	86.0 (3)	C13—C12—C11—C10	0.3 (7)
N3—Cu1—O1—C1	−104.3 (3)	C11—C12—C13—C14	0.3 (7)
N1—Cu1—O3—C8	−90.4 (3)	C12—C13—C14—C9	−1.1 (6)
N3—Cu1—O3—C8	99.6 (3)	N1—C15—C16—C17	2.0 (6)
O1—Cu1—N1—C15	−150.7 (3)	C18—C17—C16—C15	−0.1 (6)
O1—Cu1—N1—C19	38.8 (3)	C19—C18—C17—C16	−1.5 (5)
O3—Cu1—N1—C15	21.8 (3)	C20—C18—C17—C16	176.8 (3)
O3—Cu1—N1—C19	−148.8 (3)	C17—C18—C20—O5	−155.7 (4)
N3—Cu1—N1—C15	126.5 (6)	C17—C18—C20—N2	23.1 (5)
N3—Cu1—N1—C19	−44.0 (8)	C19—C18—C20—O5	22.5 (5)
O1—Cu1—N3—C21	151.1 (3)	C19—C18—C20—N2	−158.6 (4)
O1—Cu1—N3—C25	−30.9 (3)	N1—C19—C18—C17	1.3 (5)
O3—Cu1—N3—C21	−21.3 (3)	N1—C19—C18—C20	−177.1 (3)
O3—Cu1—N3—C25	156.7 (3)	C23—C22—C21—N3	0.4 (6)
N1—Cu1—N3—C21	−126.0 (6)	C24—C23—C22—C21	−1.0 (6)
N1—Cu1—N3—C25	52.0 (8)	C25—C24—C23—C22	0.4 (6)
O10—Cu2—O8—C27	−22.5 (8)	C26—C24—C23—C22	−178.1 (4)
N5—Cu2—O8—C27	91.9 (3)	C23—C24—C25—N3	0.9 (6)
N7—Cu2—O8—C27	−100.8 (3)	C26—C24—C25—N3	179.4 (3)
O8—Cu2—O10—C34	12.8 (8)	O6—C26—C24—C23	153.3 (4)
N5—Cu2—O10—C34	−102.0 (3)	O6—C26—C24—C25	−25.2 (5)
N7—Cu2—O10—C34	91.2 (3)	N4—C26—C24—C23	−26.7 (5)
O8—Cu2—N5—C41	−17.6 (3)	N4—C26—C24—C25	154.8 (4)
O8—Cu2—N5—C45	156.3 (3)	O8—C27—C28—C29	−10.3 (5)
O10—Cu2—N5—C41	152.4 (3)	O8—C27—C28—C33	168.5 (4)
O10—Cu2—N5—C45	−33.7 (3)	O9—C27—C28—C29	168.0 (4)
N7—Cu2—N5—C41	−124.1 (5)	O9—C27—C28—C33	−13.2 (6)
N7—Cu2—N5—C45	49.7 (7)	C30—C29—C28—C27	177.8 (4)
O8—Cu2—N7—C47	17.3 (3)	C30—C29—C28—C33	−1.0 (6)
O8—Cu2—N7—C51	−157.9 (3)	C28—C29—C30—C31	−0.3 (6)
O10—Cu2—N7—C47	−152.0 (3)	C27—C28—C33—C32	−177.4 (4)
O10—Cu2—N7—C51	32.8 (3)	C29—C28—C33—C32	1.3 (6)
N5—Cu2—N7—C47	124.3 (5)	F3—C31—C30—C29	−179.2 (4)
N5—Cu2—N7—C51	−50.9 (7)	C32—C31—C30—C29	1.5 (6)
Cu1—O1—C1—O2	5.2 (5)	C33—C32—C31—F3	179.5 (4)
Cu1—O1—C1—C2	−174.5 (2)	C33—C32—C31—C30	−1.2 (6)
Cu1—O3—C8—O4	−6.3 (5)	C31—C32—C33—C28	−0.3 (6)
Cu1—O3—C8—C9	172.4 (2)	O10—C34—C35—C36	−16.3 (6)
Cu2—O8—C27—O9	−9.5 (5)	O10—C34—C35—C40	161.8 (4)
Cu2—O8—C27—C28	168.8 (2)	O11—C34—C35—C36	165.3 (4)
Cu2—O10—C34—O11	15.2 (5)	O11—C34—C35—C40	−16.6 (6)
Cu2—O10—C34—C35	−163.1 (3)	C34—C35—C40—C39	−178.7 (4)
Cu1—N1—C15—C16	−172.5 (3)	C36—C35—C40—C39	−0.6 (6)
C19—N1—C15—C16	−2.2 (5)	C37—C36—C35—C34	−179.8 (4)
Cu1—N1—C19—C18	171.8 (3)	C37—C36—C35—C40	2.0 (6)
C15—N1—C19—C18	0.5 (5)	C35—C36—C37—C38	−2.4 (6)
Cu1—N3—C21—C22	178.9 (3)	F4—C38—C37—C36	−178.6 (4)
C25—N3—C21—C22	0.8 (6)	C39—C38—C37—C36	1.4 (6)
Cu1—N3—C25—C24	−179.6 (3)	F4—C38—C39—C40	−180.0 (3)
C21—N3—C25—C24	−1.4 (5)	C37—C38—C39—C40	0.0 (6)
C45—N5—C41—C42	2.0 (6)	C35—C40—C39—C38	−0.4 (6)
Cu2—N5—C41—C42	175.8 (3)	N5—C41—C42—C43	−1.7 (6)
Cu2—N5—C45—C44	−174.8 (3)	C44—C43—C42—C41	0.0 (6)
C41—N5—C45—C44	−0.6 (5)	C45—C44—C43—C42	1.4 (5)
Cu2—N7—C47—C48	−176.2 (3)	C46—C44—C43—C42	−174.2 (3)
C51—N7—C47—C48	−1.0 (5)	C43—C44—C46—O12	155.7 (4)
Cu2—N7—C51—C50	175.3 (3)	C43—C44—C46—N6	−21.1 (6)
C47—N7—C51—C50	−0.3 (5)	C45—C44—C46—O12	−19.9 (5)
O1—C1—C2—C3	−11.7 (5)	C45—C44—C46—N6	163.3 (4)
O1—C1—C2—C7	169.0 (4)	N5—C45—C44—C43	−1.1 (6)
O2—C1—C2—C3	168.6 (4)	N5—C45—C44—C46	174.8 (3)
O2—C1—C2—C7	−10.7 (6)	N7—C47—C48—C49	1.2 (6)
C1—C2—C3—C4	−179.6 (4)	C47—C48—C49—C50	−0.2 (6)
C7—C2—C3—C4	−0.3 (6)	C51—C50—C49—C48	−0.9 (5)
C1—C2—C7—C6	−179.8 (4)	C52—C50—C49—C48	177.1 (3)
C3—C2—C7—C6	0.9 (6)	C49—C50—C51—N7	1.3 (6)
C2—C3—C4—C5	0.3 (7)	C52—C50—C51—N7	−176.9 (3)
C3—C4—C5—F1	179.8 (4)	C49—C50—C52—O13	−154.6 (4)
C3—C4—C5—C6	−0.9 (7)	C49—C50—C52—N8	25.3 (5)
C7—C6—C5—F1	−179.3 (4)	C51—C50—C52—O13	23.5 (5)
C7—C6—C5—C4	1.5 (7)	C51—C50—C52—N8	−156.6 (4)
C5—C6—C7—C2	−1.4 (7)	C56—C57—N9—C53	0.1 (6)
O3—C8—C9—C10	166.5 (4)	C54—C53—N9—C57	0.4 (6)
O3—C8—C9—C14	−14.3 (5)	C56—C55—C54—C53	1.4 (6)
O4—C8—C9—C10	−14.8 (6)	N9—C53—C54—C55	−1.1 (6)
O4—C8—C9—C14	164.4 (4)	C54—C55—C56—C57	−0.9 (6)
C8—C9—C10—C11	178.5 (4)	C54—C55—C56—C58	178.5 (4)
C14—C9—C10—C11	−0.7 (6)	N9—C57—C56—C55	0.1 (6)
C8—C9—C14—C13	−177.9 (4)	N9—C57—C56—C58	−179.3 (4)
C10—C9—C14—C13	1.3 (6)	O15—C58—C56—C55	177.4 (4)
F2—C12—C13—C14	−179.8 (4)	N10—C58—C56—C55	−1.4 (6)
C9—C10—C11—C12	−0.1 (6)	O15—C58—C56—C57	−3.3 (6)
F2—C12—C11—C10	−179.6 (4)	N10—C58—C56—C57	177.9 (4)

### Hydrogen-bond geometry (Å, °)

**Table:**

*D*—H···*A*	*D*—H	H···*A*	*D*···*A*	*D*—H···*A*
N2—H2A···O18	0.86	2.10	2.929 (6)	163
N2—H2B···O4^i^	0.86	2.13	2.914 (5)	150
N4—H4A···O18^ii^	0.86	2.27	3.090 (6)	160
N4—H4B···O4^iii^	0.86	2.12	2.909 (5)	152
N6—H6A···O21^iv^	0.86	2.00	2.849 (5)	169
N6—H6B···O9^v^	0.86	2.18	2.925 (4)	145
N8—H8A···O16	0.86	2.44	3.285 (5)	167
N8—H8B···O9^vi^	0.86	2.07	2.890 (4)	160
N10—H10A···O7	0.86	2.20	3.031 (5)	163
N10—H10B···O12^vii^	0.86	2.10	2.897 (5)	155
O7—H71···O13^vii^	0.92 (3)	1.85 (3)	2.762 (4)	174 (3)
O7—H72···O14^vii^	0.81 (5)	2.02 (5)	2.824 (4)	174 (3)
O14—H141···N9	0.93 (4)	1.93 (4)	2.812 (4)	158 (4)
O14—H142···O5^viii^	0.93 (3)	1.85 (3)	2.782 (4)	177 (5)
O16—H161···O19	0.60 (4)	2.26 (3)	2.845 (6)	164 (8)
O17—H172···O6^ix^	0.73 (5)	2.21 (5)	2.887 (5)	156 (5)
O18—H182···O17^x^	0.63 (6)	2.30 (6)	2.839 (6)	145 (7)
O19—H191···O13	0.74 (5)	2.06 (5)	2.800 (6)	172 (5)
O20—H201···O18^viii^	0.77	2.07	2.611 (6)	128.
O20—H202···O15	0.64	2.14	2.710 (5)	149.
O21—H211···O2	0.91 (3)	1.91 (3)	2.807 (4)	169 (4)
O21—H212···O16^xi^	0.89 (4)	1.88 (5)	2.759 (5)	168 (5)
C4—H4···Cg9^xii^	0.93	2.81	3.555 (4)	138
C29—H29···Cg9^xii^	0.93	2.55	3.361 (5)	146

Symmetry codes: (i) −*x*, *y*−1/2, −*z*+1/2; (ii) *x*, *y*+1, *z*; (iii) −*x*, *y*+1/2, −*z*+1/2; (iv) −*x*, −*y*+1, −*z*+1; (v) −*x*+1, *y*+1/2, −*z*+3/2; (vi) −*x*+1, *y*−1/2, −*z*+3/2; (vii) *x*, −*y*+1/2, *z*−1/2; (viii) *x*, −*y*+1/2, *z*+1/2; (ix) −*x*+1, −*y*+1, −*z*+1; (x) −*x*+1, −*y*, −*z*+1; (xi) −*x*, −*y*, −*z*+1; (xii) *x*, −*y*−1/2, *z*−3/2.
